# Chemically Mediated Arrestment of the Bed Bug, *Cimex lectularius*, by Volatiles Associated with Exuviae of Conspecifics

**DOI:** 10.1371/journal.pone.0159520

**Published:** 2016-07-19

**Authors:** Dong-Hwan Choe, Hoeun Park, Claudia Vo, Alexander Knyshov

**Affiliations:** 1 Department of Entomology, University of California Riverside, Riverside, California, United States of America; 2 Department of Media & Cultural Studies, University of California Riverside, Riverside, California, United States of America; 3 Department of Biology, University of California Riverside, Riverside, California, United States of America; New Mexico State University, UNITED STATES

## Abstract

Extracts of the exuviae (cast skins) of nymphal bed bugs (*Cimex lectularius*) were analyzed for volatile compounds that might contribute to arrestment of adult bed bugs. Four volatile aldehydes, (*E*)-2-hexenal, 4-oxo-(*E*)-2-hexenal, (*E*)-2-octenal, and 4-oxo-(*E*)-2-octenal were consistently detected in the headspace of freshly shed exuviae regardless of the developmental stages from which the exuviae were obtained. Quantification of the aldehydes in the solvent extracts of homogenized fresh, 45- or 99-d aged 5th instar exuviae indicated that the aldehydes are present in the exuviae and dissipate over time, through evaporation or degradation. Microscopic observation of the fifth instar exuviae indicated that the dorsal abdominal glands on the exuviae maintained their pocket-like structures with gland reservoirs, within which the aldehydes might be retained. Two-choice olfactometer studies with the volatiles from exuviae or a synthetic blend mimicking the volatiles indicated that adult bed bugs tend to settle close to sources of the aldehydes. Our results imply that the presence and accumulation of bed bug exuviae and the aldehydes volatilizing from the exuviae might mediate bed bugs’ interaction with their microhabitats.

## Introduction

It has been long known that bed bugs (*Cimex lectularius* L.) are associated with a characteristic odor. For example, Kemper [1] described the odor associated with bed bugs as “obnoxious sweetness”. Two aldehydes that were originally identified from extracts of crushed bed bugs, (*E*)-2-hexenal and (*E*)-2-octenal, have been considered as two of the main components of the “bed bug odor” [2, 3]. Depending upon developmental stages, bed bugs produce these volatile aldehydes in two different glands. For example, nymphal bed bugs produce the compounds in dorsal abdominal glands on the third through fifth abdominal tergites, whereas adult bed bugs produce and store these aldehydes in their metathoracic scent glands [4, 5].

Based on experimental evidence so far, these two aldehydes seem to have two major functions in bed bug chemical ecology. First, (*E*)-2-hexenal and (*E*)-2-octenal function as defensive compounds or general alarm pheromones when they are released in high concentrations, causing “dispersal of assembled individuals” [6–9]. For example, relatively large amounts of these aldehydes are released when bed bugs are attacked by predators (e.g., bats or ants) [10], or disrupted with high concentrations of carbon dioxide [11], abrasive powder [12], or “unwanted” mating attempts [13, 14]. Second, several recent studies have indicated that (*E*)-2-hexenal and (*E*)-2-octenal might function as part of the aggregation pheromone blend of bed bugs. In this latter group of studies, researchers often detected relatively low concentrations of (*E*)-2-hexenal and (*E*)-2-octenal from the headspace of a bed bug colony or a paper substrate that was used to maintain bed bug colonies. For example, Siljander et al. [15] detected (*E*)-2-hexenal and (*E*)-2-octenal from aeration samples from colony jars containing 500–700 bed bugs for 3 d. Mendki et al. [16] detected these compounds from a methanol extract of a piece of filter paper substrate that was used to keep 500 tropical bed bugs (*C*. *hemipterus*) for 10 d. Gries et al. [17] reported these aldehydes by analyzing thermally-desorbed volatile compounds from a piece of filter paper that was used for keeping ≈300 bed bugs for 32 d. Based on several olfactometer studies, Siljander et al. [15] showed that (*E*)-2-hexenal and (*E*)-2-octenal function as the two most abundant aggregation pheromone components of bed bugs. Gries et al. [17] and Ulrich et al. [9] also demonstrated that (*E*)-2-hexenal and (*E*)-2-octenal are important components of the bed bug aggregation pheromone blend.

Even though the behavioral effects of these chemicals have been investigated in several empirical studies, the potential mechanisms to explain the presence of these aldehydes in the headspace and colony substrates have seldom been investigated. For example, Siljander et al. [15] stated that the exact source of (*E*)-2-hexenal and (*E*)-2-octenal detected in the colony headspace were unknown. Also, Mendki et al. [16] and Gries et al. [17] did not explicitly discuss potential reasons why these volatile aldehydes (that are produced and stored in the scent glands) were detected on the filter paper substrate that was used by the bed bug colonies.

There are several possible explanations for the presence of (*E*)-2-hexenal and (*E*)-2-octenal in the headspace odors and on the paper substrate of bed bug colonies. If the live bed bugs constantly release low concentrations of these aldehydes, this would explain the presence of the aldehydes in headspace and paper substrate of the colonies. Alternatively, frequent mating attempts by the numerous adult male bed bugs in the colony might trigger the defensive release of these aldehydes by other bed bugs. Also, anaesthetization of the colony with carbon dioxide for experimental purposes (e.g., collecting bed bugs from the colony jar) might cause the disturbed individuals to produce large amounts of these aldehydes, resulting in some adsorbing onto the colony substrate and subsequently volatilizing from the substrate. Lastly, dead bed bugs and exuviae that typically accumulate at the bottom of the colony jar could be sources of the aldehydes. Feldlaufer et al. [18] reported the presence of (*E*)-2-hexenal and (*E*)-2-octenal, along with two other oxygenated aldehydes, 4-oxo-(*E*)-2-hexenal and 4-oxo-(*E*)-2-octenal, in the excised dorsal abdominal glands of bed bug nymphal exuviae.

The current study examined whether the bed bug exuviae function as “dispensers” of the volatile pheromones, allowing the aldehydes to be slowly released from dorsal abdominal glands on the exuviae. To determine this, the study investigated: (1) if the volatile aldehydes could be detected in the headspace of exuviae, and (2) if quantities of the aldehydes in the exuviae decreased as the exuviae aged. To understand the behavioral significance of the volatile aldehydes, bed bugs’ arrestment response to sources of the aldehydes was studied in a two-choice olfactometer. The morphological characteristics of dorsal abdominal glands on the exuviae are also described. The potential significances of the volatiles associated with the exuviae on bed bug ecology are discussed.

## Materials and Methods

### Insects

Bed bugs used in these experiments came from stock colonies started from “Earl” strain individuals purchased from Sierra Research Laboratories (Modesto, CA, USA). The Earl strain was originally collected in Modesto, CA in 2007. The bed bugs were kept in screened containers, and fed with a grafting tape membrane (Aglis & Co., Ltd., Yame City, Fukuoka, Japan) feeder containing rabbit blood plus sodium citrate (Quad Five, Ryegate, MO, USA) once a week. All colonies were kept at 26°C, 35–38% RH, with a photoperiod of 12:12 (L:D) h.

### Exuviae

First instar nymphs were obtained from the eggs laid by 54 female adults that were isolated from two stock colonies on day 1 post-feeding. The females were placed in the cells (16 mm diameter by 19 mm depth) of 24-well plastic culture plates (Corning Inc., Corning, NY, USA) (two females per cell), and allowed to lay eggs for 1 wk. Once eggs were laid on the filter paper disc, the female bed bugs were removed from the plates. Once first instar nymphs hatched from the eggs, a fine paintbrush was used to move the insects to new colony vials. Six small colonies were established with approximately ≈50 first instar nymphs per container (total ≈300 insects initially). These six small colonies were used to collect 1st, 2nd, 3rd, 4th, and 5th instar exuviae.

To ensure that most individuals were fully fed, the colonies were fed with an artificial feeding system 3 times within 5 consecutive days. The exuviae that had fallen to the bottom of the vials were collected using a fine paintbrush within 9 d after the most recent feeding session. Twenty-five exuviae were collected and placed in a GC vial (2 ml, Agilent Technologies, Santa Clara, CA, USA). Each vial contained exuviae from only the same instar. The vials were sealed with a cap (9 mm in diameter, with PTFE/red silicon septa, Agilent Technologies) and kept at room temperature (25°C) until being used for analyses of the headspace volatiles. All of the chemical analyses were conducted within 7 d after the collection of exuviae. Extra exuviae were removed from the bed bug rearing vials after each session of exuviae collection.

To minimize any experimental artifacts in terms of volatile aldehyde release, two precautions were taken. First, the use of carbon dioxide gas for temporary anesthesia was avoided throughout bed bug maintenance and exuviae collection. High concentrations of carbon dioxide can stimulate bed bugs to release large amounts of volatile aldehydes from their scent glands [11, 19]. Second, the colonies were continuously monitored for the presence of adult bed bugs, and they were removed from the colonies as soon as they were discovered (either between or during the exuviae collections, before feeding). Bed bugs produce high concentrations of these volatiles when they are all confined within a small colony vial and fed simultaneously, primarily due to males’ mating attempts and defensive responses of other individuals toward unwanted mating attempts [13, 14].

### Headspace Analysis

To determine if bed bug exuviae produce volatile compounds, headspace chemicals of the exuviae were collected by solid phase microextraction (SPME). Exuviae from different nymphal instars were examined separately. Headspace volatiles of a group of 25 exuviae in a vial were collected with a SPME sampler [75 μm carboxen / polydimethylsiloxane (PDMS); Supelco, Inc., Bellefonte, PA, USA] by exposing the SPME fiber in the headspace of a glass vial through the septum cap (18-h collection). Volatile collections were conducted at 25–26°C. The protocol for volatile collection was established based on preliminary study with SPME fibers with different coating materials (e.g., 100 μm PDMS, 65 μm PDMS/DVB; Supelco, Inc.) and various collection times, optimizing the detection sensitivity for the compounds of interest.

Immediately following the collection, volatiles adsorbed on the SPME fiber were analyzed by gas chromatography– mass spectrometry (GC-MS). Electron impact mass spectra (70 eV) were taken with an Agilent 5975C mass selective detector interfaced to an Agilent 7890A gas chromatograph equipped with a DB-5 column (30 m × 0.32 mm inner diameter, Agilent Technologies). Samples were injected in splitless mode, with a temperature program of 50°C for 1 min and then 10°C min^−1^ to 280°C with 5-min hold. The temperatures of the injector and transfer line were 250°C. Helium was used as the carrier gas.

### Extract Analysis

Exuviae obtained from 5th instar nymphs (i.e., from their molting to the adult stage) were extracted to examine the chemical contents of dorsal abdominal glands on the exuviae. To determine if quantities of the chemicals decrease as the exuviae age in the open air, three groups of fifth instar exuviae were prepared and aged for different time periods before extraction. Fifth instar exuviae were collected from the nymph-only experimental colonies at 5–7 d post-feeding. The three aging periods (i.e., time period between collection of the exuviae and extraction) tested were 7, 45, and 99 d. A total of ≈50 exuviae were initially prepared per aging period by placing ≈25 exuviae into each glass vial (4-ml). For the aging process, the glass vials containing exuviae were left in a shaded area of the laboratory (22–25°C) away from ambient light without caps for corresponding periods.

The chemical contents of the dorsal abdominal glands of the exuviae were extracted by crushing a group of three exuviae with a glass tissue grinder (Potter-Elvehjem Tissue Grinder, Kimble/Kontes Glass Co., Vineland, NJ, USA) containing 0.5 ml methylene chloride. After thoroughly crushing the exuviae, the methylene chloride extract was pipetted out and filtered through a 14-cm glass pipette column containing a small glass wool plug to separate the exuviae fragments from the solvent extract. The filtered extract (≈0.5 ml) was collected in a 2-ml glass vial with graduation marks and clean methylene chloride was added to bring the volume to exactly 0.5 ml. One-microliter aliquots of the extracts were analyzed in an Agilent 7890 gas chromatograph equipped with a DB-5 column (30 m × 0.25 mm inner diameter, Agilent Technologies) and a flame ionization detector. Helium was used as the carrier gas. To avoid cross contaminations, the glass tissue grinder was washed thoroughly with acetone between different sets of samples, and new pipettes and glass wool were used for each sample. For each aging period, there were 10 replications.

### Synthetic Pheromones

Authentic standards of (*E*)-2-hexenal and (*E*)-2-octenal were purchased from Sigma-Aldrich (St. Louis, MO, USA), and 4-oxo-(*E*)-2-hexenal and 4-oxo-(*E*)-2-octenal were synthesized as previously described by Moreira and Millar [20]. For chemical identification, retention times and mass spectral data were compared between the synthetic standards and natural compounds obtained from the bed bug exuviae. To quantify the natural pheromone compounds in the exuviae, a series of external standards of known concentrations [0.12–15.63 μg/ml for (*E*)-2-hexenal and (*E*)-2-octenal; 0.12–7.50 μg/ml for 4-oxo-(*E*)-2-hexenal and 4-oxo-(*E*)-2-octenal] were prepared in methylene chloride, and 1 μl of the sample was analyzed in an Agilent 7890 gas chromatograph equipped with a DB-5 column and a FID as described above. Helium was used as the carrier gas. Calibration curves were established for each compound, and the quantity of each compound in the exuviae was estimated based on the corresponding calibration curve. To obtain quantity information per exuvia, the quantity values estimated for each sample (three exuviae combined) were divided by 3, assuming that three exuviae used to prepare a sample contributed equally to the total quantity.

### Gland Morphology

To determine the morphological characteristics of the dorsal abdominal glands on exuviae, the 5th instar exuviae were observed with a scanning electron microscope (SEM). Intact and cut exuviae (along the longitudinal centerline to cut the dorsal abdominal glands into half) were cleaned by dipping in *D*-limonene and subsequently submerged in acetone for >24 h. The washed exuviae were kept in an oven for 24 h (150°C) to remove the solvent. Stub-mounted specimens were sputter-coated using a Cressington Scientific 108 Auto device (Cressington Co., Watford, Hertfordshire, UK) with a gold-palladium mixture, and observed with a Philips XL30-FEG scanning electron microscope (working voltage 10 kV).

The dorsal tergites of 5th instar exuviae were also prepared as slice samples after embedding the exuvia sample in histological resin. Technovit^®^ 7100 embedding kit (Heraeus Kulzer GmbH, Wehrheim, Germany) was used for embedding the specimens. Pre-infiltration, infiltration, and polymerization steps were completed following the kit’s user instructions. Specimens were first embedded in a flat embedding mold (PELCO^®^ 21 Cavity EM Embedding Mold, Ted Pella Inc., Redding, CA, USA), which allowed precise positioning of the specimen. After polymerization, the blocks with the specimens were extracted and placed into a round vertical embedding mold (SPI Easy Molds^™^ LKB-2208-156, SPI Supplies / Structure Probe Inc., West Chester, PA, USA), which allowed better fixation of the blocks in the block holders (6 mm EBH-2 Block Holder). Sorvall JB-4 Microtome with glass knives was used to perform slicing, which was done in both transverse and sagittal planes with the slice thickness set to 5 μm. Slices were collected into a plastic boat filled with water, transferred onto glass slides, and put on a slide warmer. After drying, the specimens were covered with coverslips and observed using a compound microscope. A Zeiss Axioskop 2 compound microscope with a digital camera was used to obtain images of the specimens.

To determine if the dorsal abdominal gland reservoirs open externally, we examined whether a colored solvent could be taken up into the gland reservoir by submerging the exuviae in a colored solvent [*D*-limonene with Sudan Black B (Fisher Scientific)] and gently pressing and releasing the gland reservoirs with a fine probe. An excess of Sudan Black B was mixed with a small amount of *D*-limonene (1 ml), and the mixture was filtered to remove undissolved particles (Spin-X centrifuge tube filter, 0.22 μm Nylon, Corning Inc.). The surfaces of the treated exuviae were briefly washed with clean acetone, and observed under a stereomicroscope with bright-field illumination.

### Olfactometer Study

**Fig 1:**
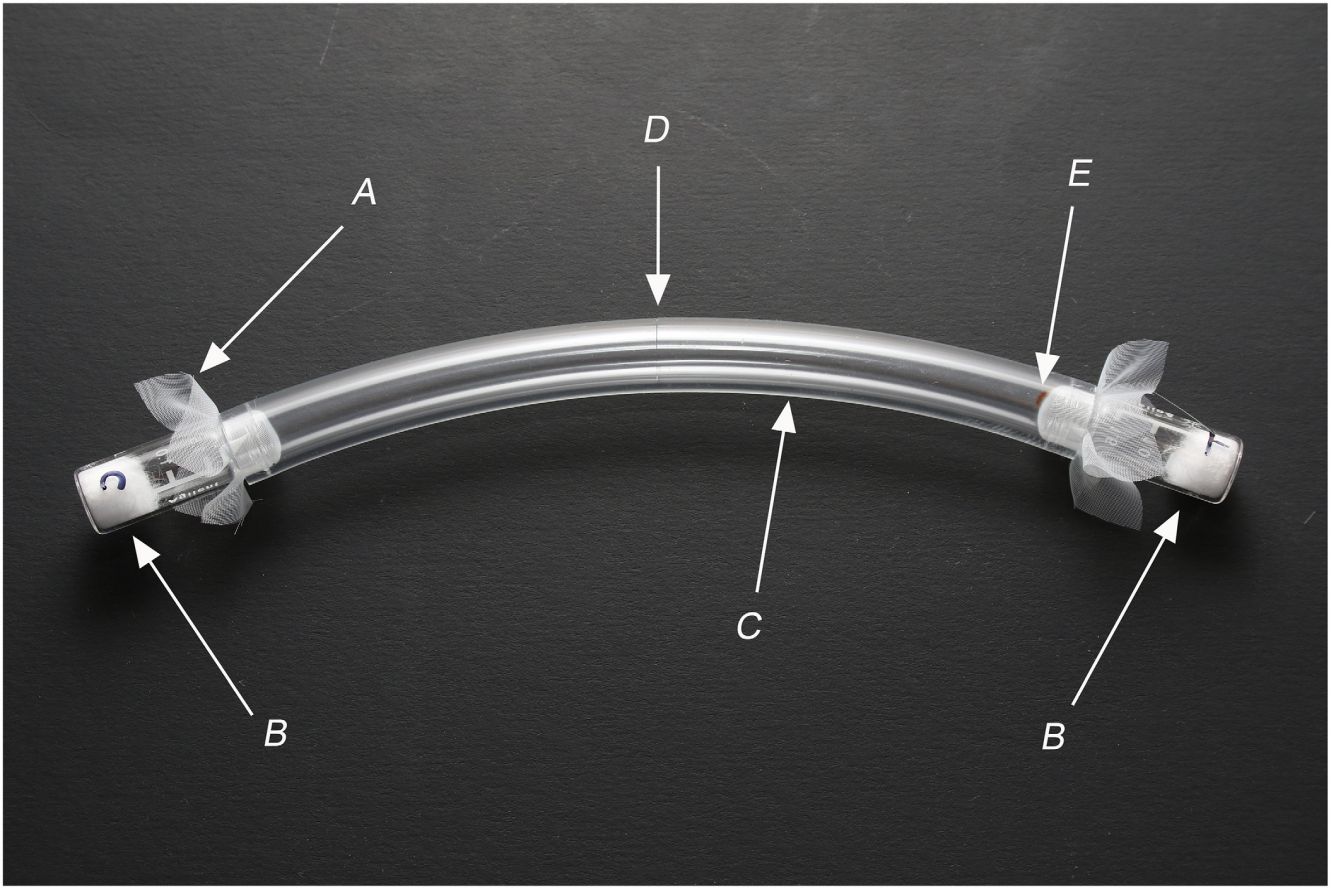
Tubing olfactometer. A piece of fine fabric screen (A) was placed before inserting vials (B) into the 15-cm PVC tubing (C) to prevent the test insect from physically contacting the contents of the vials. A small slit (D) at the center of the tubing was used to introduce a single adult bed bug into the olfactometer. The bed bug (E) was typically found on the fabric screen of the chosen vial after 18 h.

The behavioral responses of adult bed bugs to the volatile components were examined in an olfactometer (Fig 1). In particular, the study tested whether individual bed bugs settled closer to the source of the test volatiles when the compounds were released from a specific location within a novel environment (i.e., olfactometer). A 15-cm piece of flexible polyvinyl chloride (PVC) tubing (Vincon flexible PVC tubing, 9.5 mm ID, 12.7 mm OD, 1.7 mm wall thickness; Saint-Gobain Performance Plastics, Garden Grove, CA, USA) was used as the body of the olfactometer. The tubing was aerated for >15 min before use to reduce any volatile compounds in the tubing material. Glass vials (2 ml, Agilent Technologies) were attached to each end of the tubing, with a fine fabric screen on the vial openings, serving as treatment and control vials. The treatment vial served as a source of test components. The piece of fabric screen prevented bed bugs in the olfactometer body from making any physical contact with vial contents. To test exuviae as a source of volatile pheromones, 70–76 exuviae from 3rd, 4th, and 5th instar nymphs were collected in the treatment vial. Clean empty vials served as controls. For testing synthetic blends in the olfactometer, blends of test compounds dissolved in paraffin oil (40 μμl) were applied to a piece of cotton (≈50 mg) in the treatment vial. Compound ratios were adjusted to match the ratio found in a 7-d old exuviae. Vials containing a piece of cotton treated with 40 μl of clean paraffin oil were used as controls.

All of the bed bugs used in the olfactometer study were fed 13–17 d before being used in the assays, and they were collected and kept individually in culture plate cells lined with filter paper discs at least 12 h prior to the bioassay. Individual bed bugs were introduced into the tubing olfactometer through a small slit cut at the center of the tubing about 3 h before the end of photophase, and final location of the bed bug was recorded 18 h later during photophase. During this period, the olfactometer was placed on an undisturbed table in a dark room (25°C). Most bed bugs chose either treatment or control vial by the end of the trial, typically being found on the fabric screen placed over the opening of the chosen vial. In one trial (male, synthetic blend study), the bed bug was found in the middle of the tubing olfactometer without choosing either side by the end of 18-h period, and this individual was removed from further data analyses. Both sexes of adult bed bugs were tested. The study was replicated 20–32 times for each sex and the test item (i.e., exuviae or synthetic pheromone blend).

### Statistical Analyses

Simple linear regression equations were calculated by the method of least squares to relate amounts of the aldehydes per exuvia (μg, y) to the aging period (number of days, x). Quantity values (y) were log-transformed before the regression analyses because there was heteroscedasticity (i.e., standard deviation of y at any given x decreases in proportion to the value of x). The significance of the regression was tested using analyses of variance (ANOVA) with either residual mean squares or within-groups mean squares depending upon the result of lack of fit test. The lack of fit test examined if the population regression was linear [21, 22].

For the olfactometer study, the null hypothesis that adult bed bugs showed no preference for either olfactometer arm (i.e., 0.5 response rate based on the random choice) was tested using Chi-square goodness of fit tests with the Yates correction for continuity [21].

## Results

### Headspace Analysis

SPME analyses indicated that (*E*)-2-hexenal, 4-oxo-(*E*)-2-hexenal, (*E*)-2-octenal, and 4-oxo-(*E*)-2-octenal were consistently detected in the headspace of exuviae regardless of the instars from which the exuviae were obtained (Table 1). The compounds were positively identified by comparing their retention times and mass spectra with those of authentic standards.

**Table 1:** Compounds identified from headspace of exuviae obtained from nymphal instars of *C*. *lectularius* by SPME and GC-MS. A “plus” sign in the cell indicates the detection of the compound. Information within parenthesis indicates the number of positive detections out of total replications (partial detections).

Instar	Replication	Compounds			
		(*E*)-2-hexenal	4-oxo-(*E*)-2-hexenal	(*E*)-2-octenal	4-oxo-(*E*)-2-octenal
1	4	+	+	+	+ (3 of 4)
2	4	+	+ (3 of 4)	+	+ (3 of 4)
3	4	+	+	+	+ (3 of 4)
4	4	+	+	+	+ (2 of 4)
5	3	+	+	+	+

### Extract Analysis

GC-FID analyses indicated that (*E*)-2-hexenal, 4-oxo-(*E*)-2-hexenal, (*E*)-2-octenal, and 4-oxo-(*E*)-2-octenal were consistently detected in all of the solvent extract samples of 5th instar exuviae. Quantities of the compounds per exuvia were estimated based on external standard analyses with identical GC-FID conditions. Quantities of (*E*)-2-hexenal, 4-oxo-(*E*)-2-hexenal, (*E*)-2-octenal, and 4-oxo-(*E*)-2-octenal per exuvia at different aging periods are shown in Table 2.

**Table 2:** Quantities (μg, mean ± SEM, n = 10 for each aging period) of four aldehydes in a 5th instar exuvia after various aging periods.

Compound	Aging period (days after exuviae collection)		
	7	45	99
(*E*)-2-hexenal	0.32 ± 0.05	0.17 ± 0.03	0.09 ± 0.01
4-oxo-(*E*)-2-hexenal	0.49 ± 0.07	0.22 ± 0.03	0.02 ± 0.002
(*E*)-2-octenal	1.13 ± 0.15	0.67 ± 0.08	0.20 ± 0.02
4-oxo-(*E*)-2-octenal	0.17 ± 0.02	0.07 ± 0.008	0.02 ± 0.001

**Fig 2:**
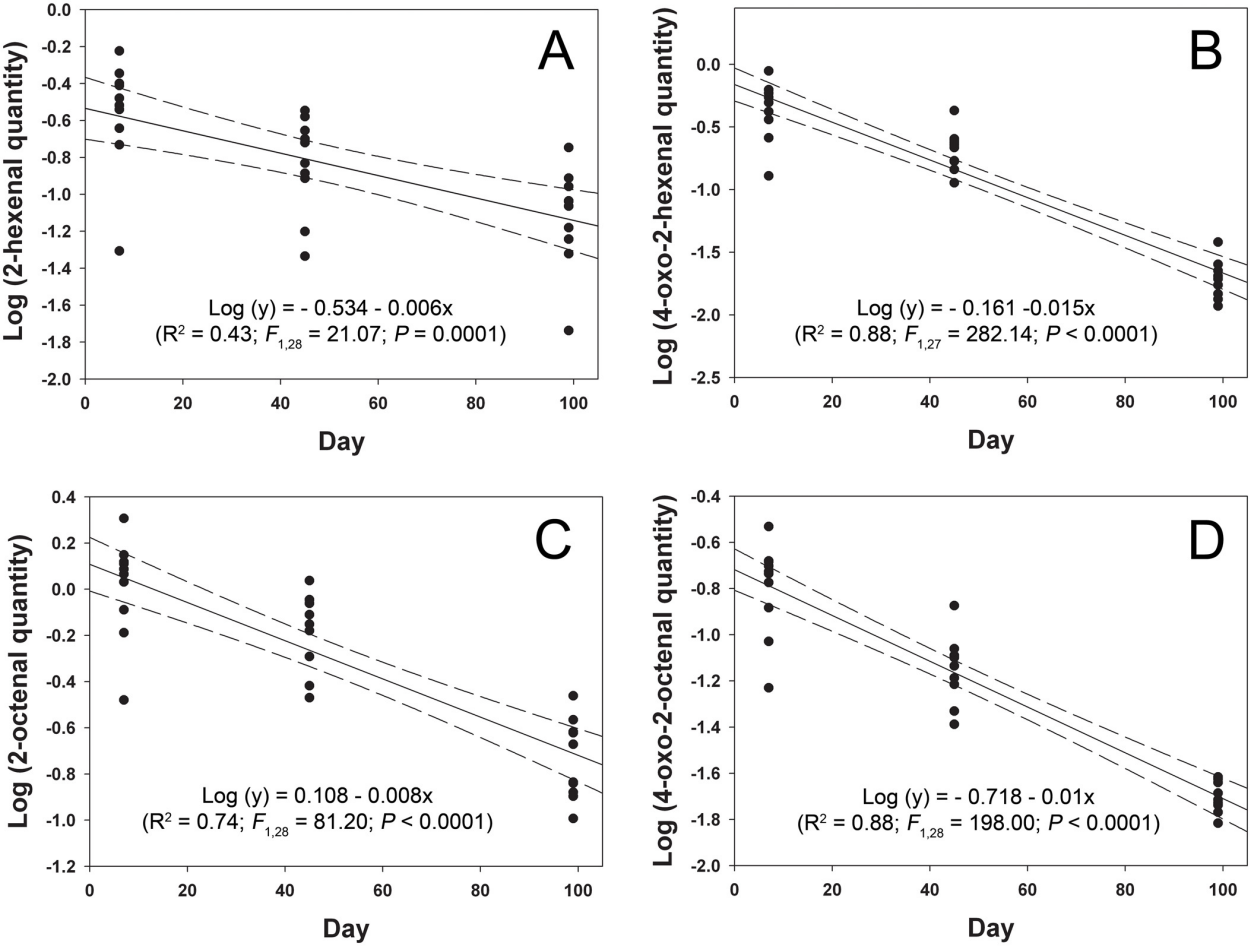
Regression lines of four aldehydes detected from exuviae of 5th instar nymphs. (A) (*E*)-2-hexenal, (B) 4-oxo-(*E*)-2-hexenal, (C) (*E*)-2-octenal, (D) 4-oxo-(*E*)-2-octenal. Units for the chemical quantity per exuvia is μg. Dotted lines indicate 95% confidence intervals.

Simple linear regression equations were calculated to relate amounts of the aldehydes per exuvia to the aging period in days. Based on the linear regression analyses, the quantities of (*E*)-2-hexenal, 4-oxo-(*E*)-2-hexenal, (*E*)-2-octenal, and 4-oxo-(*E*)-2-octenal per exuvia measured in micrograms (y) fit a log-linear model with the aging period measured in days (x) (Fig 2). Lack of fit tests indicated that the population regressions were linear for (*E*)-2-hexenal, (*E*)-2-octenal, and 4-oxo-(*E*)-2-octenal (*P* > 0.1) while the population regression for 4-oxo-(*E*)-2-hexenal was not linear (*P* = 0.003). Thus, within-groups mean square was used to test the significance of regression for 4-oxo-(*E*)-2-hexenal. The regression equations with population linearity indicated that average amounts of (*E*)-2-hexenal, (*E*)-2-octenal, and 4-oxo-(*E*)-2-octenal in an individual exuvia were reduced by approximately 1.37, 1.83, and 2.28% per day, respectively.

### Gland Morphology

**Fig 3:**
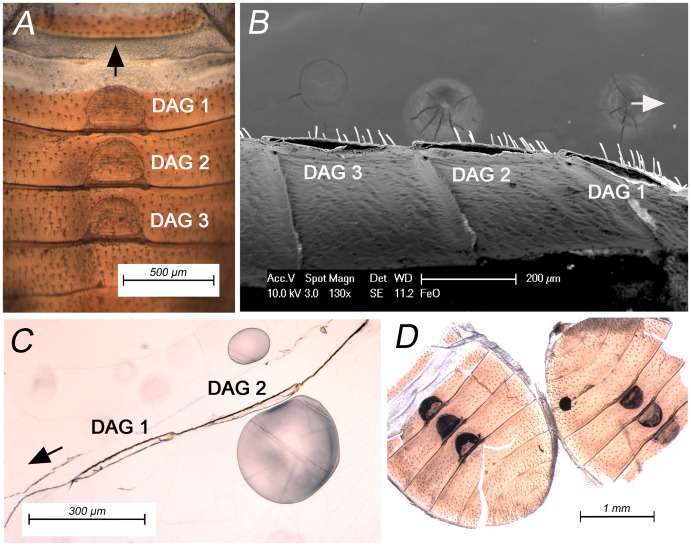
Dorsal abdominal gland morphology study with 5th instar exuviae. (A) Dorsal abdominal glands on an exuvia observed with optical dissection microscope. (B) Scanning electron microscope image showing the longitudinal cut section of dorsal abdominal glands. (C) Optical microscope image showing the cut section of dorsal area of an exuvia embedded in resin (D) Two exuviae with dorsal abdominal glands that were visualized by taking up a colored solvent into the gland reservoirs. For (A), (B), and (C), the arrow indicates the anterior direction of the exuvia. DAGs 1–3 indicate dorsal abdominal glands on the 3rd, 4th, and 5th abdominal tergites, respectively.

Three bell-shaped dorsal abdominal glands were located at the 3rd, 4th, and 5th tergites of the exuviae (Fig 3A). Based on SEM observations of the exuviae that were cut along the longitudinal centerline, it was evident that the dorsal area of the exuviae contained three reservoirs associated with dorsal abdominal glands (Fig 3B, three hollow spaces). Optical microscope observation of the slice samples obtained from the wax-embedded exuviae also supported the presence of dorsal abdominal gland reservoirs on the tergites of exuviae (Fig 3C). The black colored solvent was able to penetrate the dorsal abdominal gland reservoirs of intact exuviae, indicating that there were openings and channels connecting the internal space of the gland reservoirs to the cuticular surface (Fig 3D).

### Olfactometer Study

**Fig 4:**
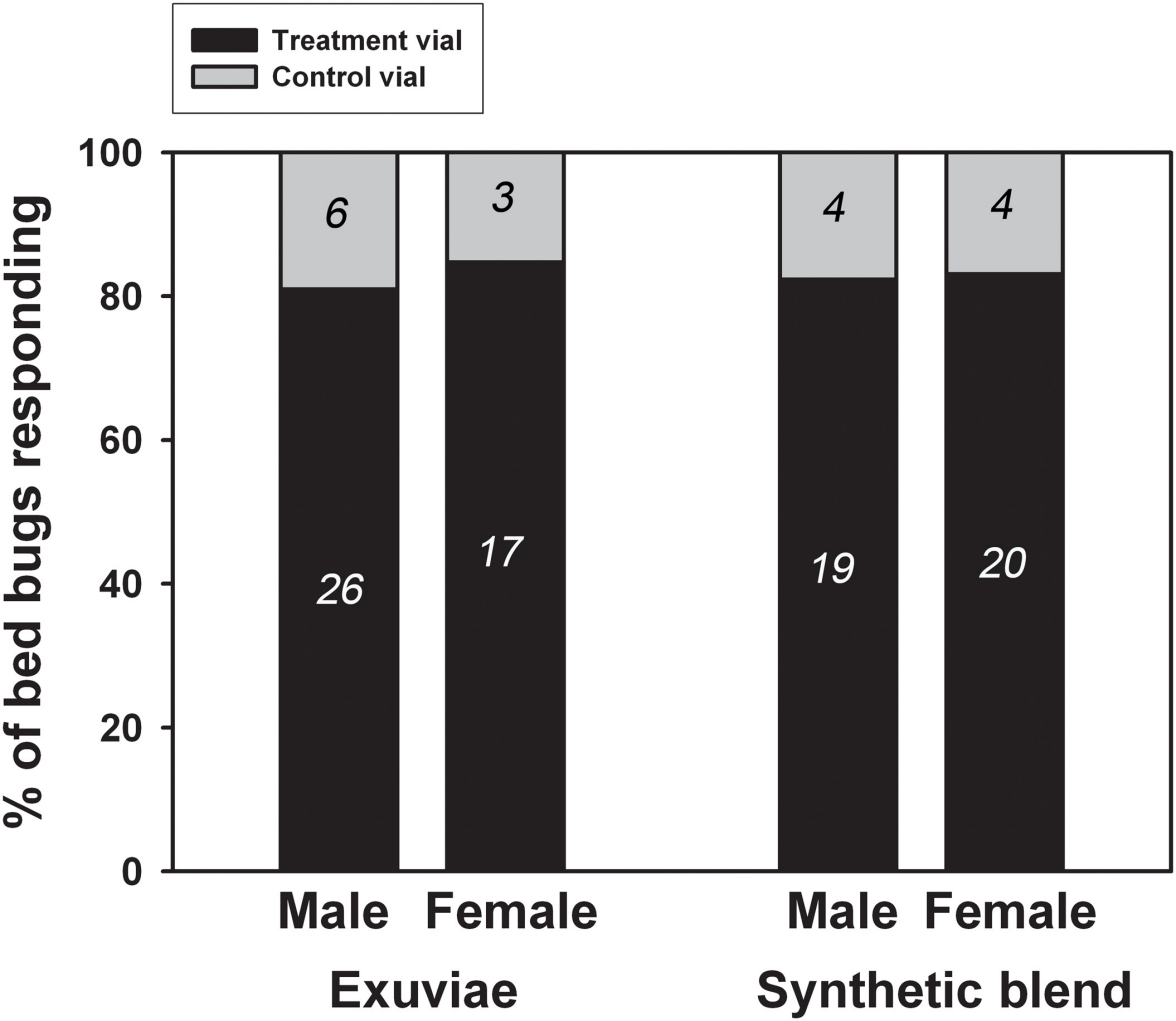
Olfactometer study with two different sources of aldehyde pheromones. For assays with the exuviae, the treatment vial contained 70–76 exuviae from 3rd, 4th, and 5th instar nymphs. For assays with the synthetic aldehydes, the treatment vial contained a small piece of cotton that was treated with 40 μl of paraffin oil containing the synthetic aldehyde blend. Control vials were either left empty (exuviae study) or contained a small piece of cotton treated with clean paraffin oil (synthetic blend study). Numbers in the bar show the number of individuals found on either treatment or control vials at the end of 18-h assay. See text for statistical analyses.

When left as individuals in the olfactometer over 18 h, adult bed bugs showed significant arrestment at the source of the volatile compounds. When the exuviae were used as a source of volatiles, 26 of 32 (81.3%) male bed bugs were found on the treatment vial of the olfactometer (*χ*^2^ = 11.28, *P* < 0.001). In the same experiment, 17 of 20 (85%) female bed bugs were found on the treatment vial (*χ*^2^ = 8.45, *P* = 0.004) (Fig 4, two bars on left).

To test a synthetic blend of the aldehydes, 40 μl of paraffin oil containing known amounts of the aldehydes [60 ng (*E*)-2-hexenal, 100 ng 2-oxo-(*E*)-2-hexenal, 225 ng (*E*)-2-octenal, and 30 ng 4-oxo-(*E*)-2-octenal] was pipetted onto a piece of cotton placed in a vial. The ratio of compounds in the synthetic blend (1:1.7:3.8:0.5) was based on the ratio of these compounds found in 7-d aged 5th instar exuviae (1:1.5:3.5:0.5) (see Table 2). The total amount of compounds used in a treatment vial was approximately one-fifth exuvia equivalent. The control vial received 40 μl of clean paraffin oil only. When the cotton ball with the synthetic blend of aldehydes was used as the source of volatiles, ≈83% (19 of 23 for male, *χ*^2^ = 8.52, *P* = 0.004; 20 of 24 for female, *χ*^2^ = 9.38, *P* = 0.002) of individuals were found on the treatment vial for both male and female bed bugs (Fig 4, two bars on right).

## Discussion

Four aldehydes [(*E*)-2-hexenal, 4-oxo-(*E*)-2-hexenal, (*E*)-2-octenal, and 4-oxo-(*E*)-2-octenal] were consistently detected in the headspace of bed bug exuviae regardless of the instars from which the exuviae were obtained. Because the exuviae were collected from the bottom of small colonies that were comprised of nymphs only, the compounds detected in the headspace of exuviae likely originated from the exuviae themselves, rather than from contamination associated with alarm / defensive responses of live bed bugs to mechanical (e.g., adult male’s mating attempts) or chemical (e.g., anesthesia with carbon dioxide) disturbances.

The extraction study with 5th instar exuviae indicated that freshly shed exuviae contained substantial amounts of aldehydes (range 0.17–1.13 μg per exuvia), and the amounts decreased with age. This information, together with the presence of the same aldehydes in the headspace of exuviae, indicates that these aldehydes slowly volatilize from the dorsal abdominal glands of the exuviae. Feldlaufer et al. [18] reported the presence of (*E*)-2-hexenal, 4-oxo-(*E*)-2-hexenal, (*E*)-2-octenal, and 4-oxo-(*E*)-2-octenal from the pentane extract of crushed dorsal abdominal glands excised from bed bug exuviae (4th and 5th instars). The presence of dorsal abdominal gland contents in the shed exuviae is known from several other species of heteropterans [23–26], and this is likely to be a general phenomenon among many species in the order Heteroptera [23]. However, it is not known whether the volatilization of dorsal abdominal gland contents from the exuviae also occurs in other heteropterans.

What morphological characteristics of the dorsal abdominal glands on the exuviae would make the retention and controlled release of the glandular compounds possible? Microscopic observations of 5th instar exuviae indicated that the exuviae retain the dorsal abdominal gland reservoirs, which are shaped like small “pouches”. Based on observations of nymphal bed bugs, Künckel [4] described the dorsal abdominal glands as “inflated small bags or sacs, with an outline which mimics the shape of a melon-glass”. Because cuticle of the integument is continuous with the invaginated cuticle which lines the interior of the gland [4], it is likely that the glandular contents would be shed along with the exuviae. The study with colored solvent indicated that the gland reservoirs on exuviae open externally, probably through pores. Künckel [4] described these small openings of dorsal abdominal glands as buttonhole-like orifices located on either side of the median line, being arranged transversely at the margin of the third, fourth, and fifth tergites (they were erroneously described as first, second, and third tergites) just over the line of separation of the segments. Overall, these evidences support the notion that the compounds inside the dorsal abdominal gland reservoirs are retained in the exuvia, and the volatile compounds in the reservoirs slowly evaporate through the small orifices.

Behavioral assays indicated that adult bed bugs are likely to settle near the fresh exuviae or pieces of cotton impregnated with a synthetic blend even without physically contacting the volatile sources. Olfactory sensillae on bed bug’s antennae are known to respond to airborne (*E*)-2-hexenal and (*E*)-2-octenal [8]. However, the current results appear to conflict with those of Gries et al. [17], who reported that physical contact is necessary to cause bed bug’s arrestment towards fresh or aged (2-month old) exuviae within a two-choice olfactometer. Nevertheless, Gries et al. [17] reported that (*E*)-2-hexenal and (*E*)-2-octenal are among the essential components of the pheromone mixture to cause attraction of bed bugs based on olfactometer trials with synthetic blends. Because the amounts of the aldehyde in the exuviae decrease over time, Gries et al. [17] and the current study might not be directly compared unless the age of the exuviae can be standardized. For example, the number and age of the exuviae (i.e., time between molting and use of the exuviae for the assay) were not specified for the olfactometer trials in Gries et al. [17]. Other differences in the olfactometer assay design (i.e., dimension) also might have resulted in different concentrations of the volatile compounds within the olfactometer, affecting the behavioral responses of the bed bugs. (*E*)-2-Hexenal and (*E*)-2-octenal are known to influence bed bugs’ behavior in a concentration-dependent manner [9, 15]. In addition to the absolute quantity of the compounds in the odor source, slow and controlled release of the compounds also might be critical to cause attraction / arrestment of bed bugs to the odor source. For example, Levinson and Bar Ilan [12] showed that paper discs scented with either (*E*)-2-hexenal or (*E*)-2-octenal or with a mixture of both aldehydes in various amounts and proportions did not elicit any aggregation of male or female bed bugs. Also, it is possible that all four major aldehydes must be present in the natural ratio to cause the maximum aggregation response.

The current study only examined the effect of four volatile aldehydes on bed bug arrestment. However, under natural conditions, bed bugs that are attracted by the volatile aldehydes are likely to contact the exuviae eventually, and then other less volatile compounds on the exuviae might further contribute to the long-term arrestment of bed bugs closer to (or on) the exuviae. Domingue et al. [27] reported that adult males were arrested on filter paper discs treated with methylene chloride extracts of 5th instar exuviae, from which dorsal abdominal glands, fecal contaminants, and hind gut contents were removed prior to the extraction. It is possible that cuticular lipids on the exuviae might be detected by bed bugs via contact chemoreception. Other compounds that are often associated with the exuviae, such as fecal substances and hindgut contents (hindgut contents are typically shed with the exuvia) also might be detected by bed bugs, further facilitating aggregation in close proximity to exuviae.

Bed bugs’ tendency of settling near conspecific exuviae might have important implications for their biology in their “natural” habitats. Bed bug harborages in residential settings can accumulate numerous exuviae from developing bed bugs. In general, bed bugs are believed to typically return to these harborages after feeding forays, forming dense aggregations, where eggs and fecal materials also accumulate [5, 6]. Attraction / arrestment to the source of aldehyde pheromones would allow the bed bugs to direct their movement towards existing harborages where other conspecific individuals are developing and producing a large number of exuviae. In addition to providing information associated with the established harborage sites, the aldehydes emanating from accumulated exuviae might play a role in modifying biological environments in bed bug harborage sites. For example, Ulrich et al. [28] reported that (*E*)-2-hexenal and (*E*)-2-octenal inhibit growth of an entomopathogenic fungus targeting bed bugs by direct contact and via indirect exposure (‘‘fumigation”), indicating these compounds may play a role in disinfecting the microenvironment of bed bug harborages. Based on research on several species of alydid, coreid, and pentatomid bugs, Noge et al. [29] and Noge [30] reported that 4-oxo-(*E*)-2-hexenal, (*E*)-2-hexenal, and (*E*)-2-octenal showed dose-dependent antibacterial activities against four bacterial species. The potential function of volatile aldehyde pheromones in shaping microbial environments associated with bed bug harborages warrants further investigation.

In the event of severe infestations with bed bugs, the number of exuviae accumulating at the harborage site can be extremely large. In such cases, especially within a small room, concentrations of the volatile aldehydes from the exuviae can be high enough to be detected by analytical methods. For example, by using solid phase microextraction (20 min– 3.5 h collection time), Eom et al. [31] detected (*E*)-2-hexenal and (*E*)-2-octenal from indoor air of rooms infested with 200–1,000 bed bugs. The compounds were not detected from control samples that were collected from kitchen (no bed bug infestation). Even though it would be impossible to determine if the exuviae was the primary source of the volatile aldehyde pheromones detected from the indoor air, it would be reasonable to assume that the exuviae would be, at least in part, responsible for the presence of the aldehydes in the indoor air. The airborne aldehydes’ potential long-term impacts on human residents warrant further research. (*E*)-2-octenal is known to have a cytotoxic effect on murine bone marrow stromal cells [32], and neurotoxic effects on *Drosophila melanogaster* [33]. Topical application of 4-oxo-(*E*)-2-hexenal has neurotoxic effects on house crickets [30].
